# The Phosphorus-Iron Nexus: Decoding the Nutrients Interaction in Soil and Plant

**DOI:** 10.3390/ijms25136992

**Published:** 2024-06-26

**Authors:** Xingqi Yang, Chang Liu, Cuiyue Liang, Tianqi Wang, Jiang Tian

**Affiliations:** Root Biology Center, State Key Laboratory for Conservation and Utilization of Subtropical Agro-Bioresources, College of Natural Resources and Environment, South China Agricultural University, Guangzhou 510642, China; yangxingqi2@stu.scau.edu.cn (X.Y.); lc0107@stu.scau.edu.cn (C.L.); liangcy@scau.edu.cn (C.L.); jtian@scau.edu.cn (J.T.)

**Keywords:** phosphorus-iron interaction, soil properties, microorganism, root morphology, root exudate, hormone, photosynthesis

## Abstract

Phosphorus (P) and iron (Fe) are two essential mineral nutrients in plant growth. It is widely observed that interactions of P and Fe could influence their availability in soils and affect their homeostasis in plants, which has received significant attention in recent years. This review presents a summary of latest advances in the activation of insoluble Fe-P complexes by soil properties, microorganisms, and plants. Furthermore, we elucidate the physiological and molecular mechanisms underlying how plants adapt to Fe-P interactions. This review also discusses the current limitations and presents potential avenues for promoting sustainable agriculture through the optimization of P and Fe utilization efficiency in crops.

## 1. Introduction

Phosphorus (P) is one of the essential mineral nutrients for plant growth and plays a critical role in the formation of organic molecules essential for plant development, including DNA, RNA, ATP and phospholipids [1]. However, agricultural production often faces problems such as suboptimal P fertilizer use efficiency and reduced soil P effectiveness, which result in plant P deficiency [2]. As a crucial micronutrient for plants, iron (Fe) is vital for the synthesis of proteins involved in photosynthesis and respiration [3]. Although Fe is the most abundant metal element in soil, its bioavailability is often low, making Fe deficiency a common occurrence in plants [4]. There is a complex interplay between Fe and P in both soil and plant, with profound effects on soil properties and plant growth [5,6]. For example, in acidic conditions, Fe is easily bounded with phosphate (Pi) to form Fe-P complex, reducing availability of both Pi and Fe in soils [7]. In plants, interaction of P and Fe influence vary of biological processes, such as Fe accumulation in apoplast inhibits primary root elongation of *Arabidopsis thaliana* under P deficiency [8], suggesting that the Fe-P interaction in plants contributes to regulate strategies of plant adaptation to nutrients deficient environments. Therefore, elucidating the mechanisms and identifying the modulating factors of the P-Fe nexus within both soil and plant systems is pivotal for advancing understanding of soil nutrient cycling and enhancing the efficiency of plant nutrient utilization.

In this review, we summarized recent breakthroughs in the interplay of P and Fe, particularly focusing on the regulation of soil properties and microorganism on Fe-P complexes formation in soil, as well discussed the intricate modulation of Fe-P interaction on network of root morphology and physiological functions in plants. Our works intend to clarify mechanisms associated with Fe-deficient and P-deficient disorders for devising strategies conducive to sustainable agriculture.

## 2. P-Fe Interactions in Soil

**Figure 1:**
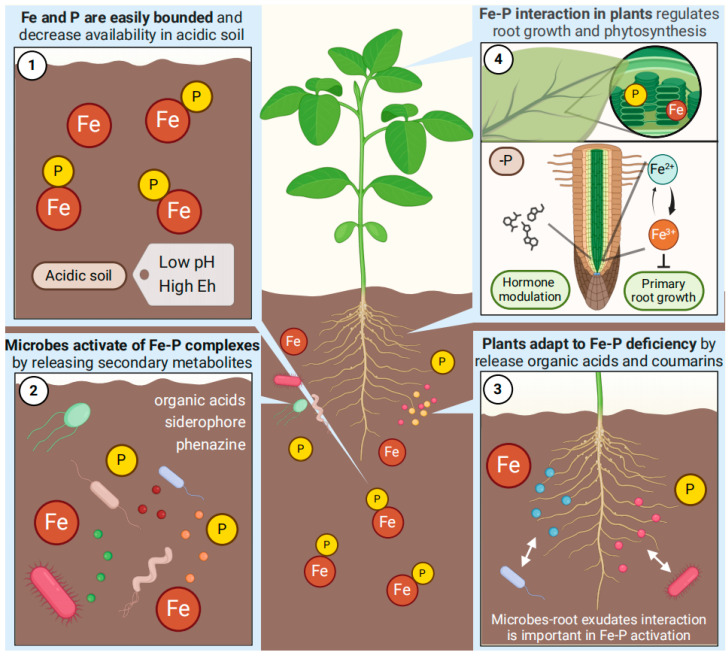
Schematic overview of the Fe-P interactions in plants and soils. 1. Fe and P are essential nutrients for plants, and their interactions participate in various biological processes in plants and soils. Fe is readily bounded to P and forms insoluble Fe-P complexes, thereby reducing the availability of Fe and P in acidic soil. 2. In the rhizosphere, microorganisms have been demonstrated to activate Fe-P complexes via secreting organic acids, siderophores, and redox substances (phenazine) into soil. 3. Furthermore, in response to Fe-P deficiencies, plants also release organic acids and redox substances (coumarins) to enhance the bioavailability of Fe and P. The interaction between microorganisms and root exudates may be crucial in promoting Fe-P activation. 4. In addition, the interaction of P and Fe exhibits intricate regulation in root morphology, hormone metabolism, and photosynthesis in plants. The mechanisms of Fe-P interaction in plants and soils have the potential to promote sustainable agriculture by enhancing P and Fe utilization efficiency in plants.

In most soils, Fe oxide is the principal Fe form, and the biogeochemical cycling of Fe based on the redox transformation between Fe^+2^ and Fe^+3^. Due to its redox-sensitive property, Fe often chemically reacts with Pi, then forms insoluble Fe-P complex via ligand exchange and surface precipitation, especially in acidic soil where Fe is more active (Figure 1) [9,10]. The surface of Fe-P complexes is then coated with a hydrated Fe oxide layer, culminating in the creation of a sealed state, further decreases bioavailability of P and Fe [11]. In addition, the inositol bounded with Pi groups on surface could be attracted to Fe oxides with positively charged colloidal surface and forming Fe-inositol-P complexes, which is firmly preserved in soil due to its stability and the resistance to phytase hydrolysis [12]. Hence, the fixation of Pi by Fe diminishes both Fe and Pi availability in soil and decelerates their biogeochemical cycling.

### 2.1. The Influences of Soil Properties on P-Fe Interactions

The forms and interaction of Pi and Fe could be regulated by physicochemical properties of soil, including soil pH, redox potential (Eh) and humic substances. Particularly, in acidic conditions, Fe oxides in soil might be the primary P-adsorbing phases [13,14]. Elevating soil pH appropriately could enhance P desorption via decreasing adsorption sites and increasing the electrostatic repulsion between clays and Pi [10,15]. The combination of P and Fe caused by low pH has become a global problem to be solved [16,17]. Especially in acidic soil in southern China, the low availability of soil Pi leads to excess P fertilizer application in farmland, which, however, causes P surplus and exacerbate soil quality deterioration [18]. The overaccumulation of P in soil, in turn, result in a reduction in the effectiveness of Fe [19]. Hence, promoting sustainable agricultural development in acidic soil requires augmenting soil P availability, especially promoting the desorption of insoluble Fe-P.

The variabilities of Eh in soils could fluctuate the biogeochemical cycling of P and Fe [20]. First, soil Eh controls the form and bioavailability of Fe [20]. The reduction condition in soil is enhanced with a decrease in the soil Eh, which induces the transformation of Fe^3+^ to Fe^2+^ and the dissolution of Fe oxides, ultimately resulting in the release of adsorbed or coprecipitated P from the Fe-P complex [21]. Conversely, elevated Eh values result in the oxidation of Fe^2+^ into Fe^3+^, thus facilitate the formation of Fe-P complex [22]. Additionally, in reduction layer of soil, Fe^3+^-oxyhydroxides can be reductively dissolved. The radial O_2_ loss (ROL), oxygen (O_2_) leakage by plant roots when adapting to low-O_2_ conditions, could drive soluble Fe^2+^ toward the root surface, which is then spontaneously oxidized and forms Fe plaques consisting of Fe hydroxides, including ferrihydrite, goethite, and siderite [23]. Fe plaques are primarily observed in proximity to mature roots, which have a negative impact on P supply to plants due to their surface chemical activity and high specific surface area [24]. It is noteworthy that the quantity of Fe plaques determines their role as an inhibitor or reservoir for soil P. Although the Fe plaque-P co-precipitates have the potential to limit the effectiveness of P, moderate Fe plaque-P may also serve as a safe haven for P, which may be dissolved and released as available Pi and Fe by secondary metabolites released by plants and microorganisms [7].

The humic substances act to retard the formation of the Fe-P complex via binding cations and the sites of precipitation and crystallization, thereby increase the solubility of P in soils [25,26]. P can be absorbed by humic-Fe complexes to form ternary humic-Fe-P complexes, which exhibit a maximum P sorption that is significantly higher than that of Fe oxides [27]. And the connection between humic substances and P primarily bases on Fe bridges in a labile form which may be easily desorbed [28]. Moreover, humic-Fe complexes exhibit a dissolution of organic P in soil, as evidenced by their higher absorption capacity for phytate compared to Fe-oxides [29]. Collectively, humic-Fe-P complexes constitute a significant pool of soil P for utilization by plants.

### 2.2. The P-Fe Interaction in Soil Affected by Microorganisms

Microorganisms are pervasive in soil, exerting a pivotal influence on mineral nutrient cycling within ecosystems and functioning as intermediaries in the communication between plants and soil [30]. P and Fe are essential mineral nutrients for microbial growth. To activate insoluble Fe-P complexes in the environment, microorganisms enhance the availability of P and Fe via the release of secondary metabolites, including organic acids, siderophores, and redox substances (Figure 1 and Table 1) [31,32].

In P-limited and Fe-limited soils, the accumulation of microorganism with organic acids secreting capacity has been observed (Table 1) [33,34,35,36]. The secretion of organic acids by microorganisms facilitates the reductive dissolution of Fe minerals, prevents the aggregation of Fe oxide crystals, results in liberation of P and Fe from Fe-P complexes [34]. Oxalic acid, citric acid and gluconic acid are the primary organic acids released in response to Pi and Fe starvation [10,35,36]. Beneficial microorganisms, such as arbuscular mycorrhizal fungi (AMF), have been shown to liberate inorganic P [37] and organic P [38] bound to Fe oxides via the release of significant amounts of organic acids [39], thereby supply sufficient P and Fe for plants [40,41,42,43]. However, a contrasting opinion demonstrates that organic acid could barely solubilize the Fe-P complex due to the high pKsp and low dissolution rates of the Fe-P complex [44,45]. Consequently, certain microorganisms may activate Fe-P complexes via distinct mechanisms, such as chelating and reducing, rather than releasing organic acids [46,47].

Siderophores, a class of secondary metabolites secreted by microorganisms under Fe deficient conditions, exhibit a high capacity for efficiently activating and chelating Fe [44,48,49]. It is noteworthy that the secretion of siderophores by microorganisms is not only induced by low-Fe conditions but also influenced by low-P conditions [50]. The abilities of microorganisms to release P and produce siderophores are consistently distinguished in numerous studies. In fact, in acidic soils with high amounts of Fe-P complexes, the release of insoluble Fe by siderophore concurrently enhances the bioavailability of P (Table 1) [31,51,52,53]. The dissolution of Fe-P complexes via siderophore could be traced back to a study from 40 years ago, in which two siderophores (i.e., desferrioxamine-B, desferriferrichrome) were demonstrated to facilitate the diffusion of Fe and P in low-pH soil [54]. This reinstated P-solubilization from Fe-organic P complexes by siderophore had also been identified in recent work [55]. Furthermore, a *Streptomyces* sp. CoT10 exhibited a high solubility for FePO_4_, which was directly mediated by its siderophores, thereby enhanced P accumulation in plants [56]. Collectively, siderophore-secreting microorganisms represent a crucial pathway for the promotion of Fe and P solubilization and availability in acidic soils.

Some microorganisms are capable of secreting reductive substances to reduce Fe^3+^ into Fe^2+^, thus inhibits the formation of Fe oxides and averts Fe-P fixation (Table 1) [57]. Phenazine, a secretion of microorganisms with reducibility, is found to be highly exudated under low-P conditions [58] and mediates specific chemical reactions leading to the dissolution of Fe oxides in soil [59,60]. A recent study has been demonstrated that phenazine secreted from *Pseudomonas* could enhance the bioavailability of P in sediment consisting of Fe oxide-P complexes [61]. In P-limited condition, the production of phenazine in *Pseudomonas* might be positively regulated by phoB, a cytoplasmic response regulator that responds to P starvation [61,62]. Further investigation is required to determine the influence of other reductive substances, such as flavins, on the activation of insoluble Fe-P complexes. It is notable that certain microorganisms possess their own reducing or oxidizing abilities, which are mediated by electron transport and transfer or oxidases and reductases. These progresses facilitate the conversion of Fe^2+^ and Fe^3+^ into each other and regulate the combination of Fe oxides colloids and PO_4_^3−^ [63,64,65,66]. Modulating the ratio of oxidizing bacteria to reducing bacteria is also a crucial aspect in enhancing the effectiveness of Fe and P in soil. In addition, the secretion of phytase represents another pathway by which microorganisms activate insoluble Fe-P complexes in soil [67]. Overall, microorganisms play a multifaceted role in the liberation of unavailable Fe-P complexes in soils, thereby contribute significantly to the biogeochemical cycling of Fe and P.

**Table 1:** Mechanisms of microorganisms in activating insoluble Fe and P in soil.

Mechanism	Function	Bacteria or Substance	References
Release organic acids	In acidic environment, secrete oxalic acid in response to insoluble P, such as goethite (P-Fe oxides)	*Aspergillus niger*	[10,36]
	Low-P soil promotes P-solubilizing bacteria accumulation and organic acids secretion	P-solubilizing bacteria	[33]
	Release citric acid to adapt to acidic environment	*Aspergillus niger*	[35]
	Dissolve P from goethite via secreting low-molecular-weight organic acids, enhance P content of *Solanum lycopersicum*	*Rhizophagus irregularis*	[39]
Siderophore secretion	Solubilization of P from Fe-phosphates at acid pH	desferrioxamine-B and desferriferrichrome	[54]
	Solubilize P from Fe-phytate complexes	Siderophore-secreting bacteria	[55]
	Activate insoluble Fe-P complexes in rhizosphere, enhance P and Fe in *Camellia oleifera*	*Streptomyces* sp. CoT10	[56]
Redox ability	Release P from Fe-rich sludge via reducing Fe^3+^ to Fe^2+^	Fe-reducing bacteria	[57]
	Release phenazine-1-carboxamide to promote Fe reduction and dissolve Fe (hydr)oxides	*Pseudomonas chlororaphis*	[59]
	Phenazines reductively dissolve ferrihydrite and hematite the reaction rate is increases as the pH decreases	Pyocyanin, phenazine-1-carboxylate, phenazine-1-carboxamide and 1-hydroxyphenazine	[60]
	Release phenazine to reduce Fe^3+^ to Fe^2+^, solubilize P from phosphate hydrous ferric oxides and marine sediments	*Pseudomonas aeruginosa*	[61]
	Increase Fe-P in sediment and oxidizing Fe^2+^ to Fe^3+^	Fe-oxidizing bacteria	[65]

## 3. Interactions of P-Fe in Plants

In responding to P or Fe deficiencies, plant employ multiple physiological and molecular mechanisms, including elevating nutrient uptake efficiency via modulating root morphology and liberating insoluble nutrients by release of exudates [68]. Intricate interactions between P and Fe are also exhibited within plant, especially in regulating root growth, metabolism of exudates and hormone, as well maintaining nutrients homeostasis [69,70,71].

### 3.1. Fe uptake Process Influenced by P

Plants have evolved two strategies for Fe uptake [72]. In strategy I, plants reduce soil Fe^3+^ to Fe^2+^ via the FERRIC REDUCTASE OXIDASE (FRO) [73] and subsequently take up Fe^2+^ into the roots via the IRON-REGULATED TRANSPORTER1 (IRT1) [74]. Additionally, under low Fe stress, strategy I plants can secrete compounds with redox activity, such as coumarins, to increase Fe availability in the soil [75,76]. In strategy II, plants could secrete mugineic acids (MAs) family phytosiderophore to chelate Fe in the soil, then the MA-Fe complexes are transported into the roots via the YELLOW STRIPE (YS) or YELLOW STRIPE LIKE (YSL) transporters [77,78].

Interestingly, under Fe-limited conditions, low-P promote Fe accumulation in plant roots [79,80,81]. Meanwhile, it is found that increasing external P concentration inhibits Fe uptake and translocation [82,83], exacerbating chlorosis induced by low-Fe [84,85]. In addition, in P-deficient condition, Fe content was not reduced in *irt1* mutants compared to wild type, suggesting that Fe uptake under P deficiency might be independent of IRT1 function [86]. In rice (*Oryza sativa* L.), the expression of genes involved in Fe uptake has been shown to be suppressed under P deficiency [87]. Conversely, the upregulation of *VACUOLAR IRON TRANSPORTER* (*VIT*) by P deficiency resulted in Fe accumulation in root vacuoles and cell walls [88], suggesting that the negative regulation of genes involved in Fe uptake may be feedback for Fe accumulation in P-deficient plants [79,88]. Although Fe deficiency also leads to P accumulation in plants [89], P deficiency is considered to have a more pronounced effect on Fe homeostasis than Fe deficiency on P homeostasis [80].

### 3.2. The Fe-Mediated Root Development Response to Low-P

In plants, roots serve as the primary organ for nutrient uptake. In response to P deficiency, plants modify root morphology, including reducing primary root (PR) elongation and increasing root hair density [90]. The maintenance of Fe homeostasis plays a pivotal role in the response to P deficiency (Figure 1). Reduction of Fe concentration alleviates the inhibition of PR elongation induced by P deficiency [91,92,93,94]. Conversely, excessive Fe supplementation exacerbated the growth inhibition of PR under P-deficiency [95,96]. The growth limitation of PR induced by low-P is primarily attributed to the deposition and valence state of Fe in the apoplast of stem cell niche and cortical cells in elongation zone [97,98,99,100].

**Figure 2:**
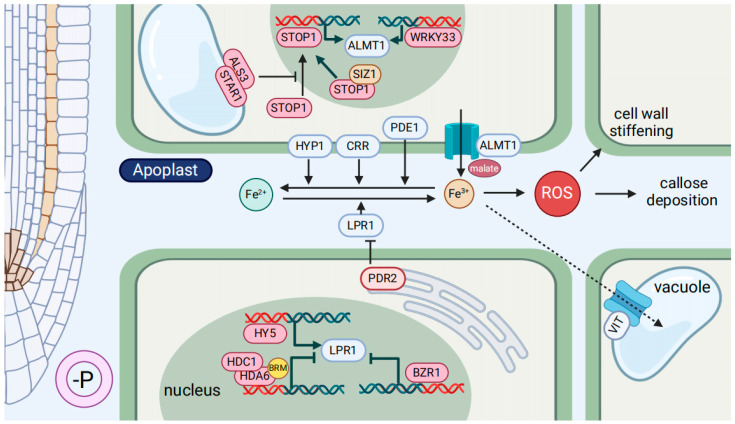
The network of Fe in the apoplast modulates primary root growth under P starvation. In P deficiency, Fe is accumulated in the apoplast of stem cell niche and elongation zone cortical cells (yellow part of root on the left side of diagram). The accumulation of Fe triggers the production of reactive oxygen species (ROS), which induces cell wall stiffening and callose deposition, thereby suppresses primary root elongation. This process necessitates the participation of LPR1 in the oxidation of Fe^2+^ and the involvement of CRR, HYP1, and PDE1 in the reduction of Fe^3+^. BZR1 and the BRM-mediated recruitment of the HDC1-HDA6 complex to the LPR1 loci both repress *LPR1* transcription, while HY5 elevates the expression of *LPR1*. PDR2 negatively regulates the accumulation of apo-Fe under low-P conditions by restricting the actions of LPR1. The secretion of malate into the apoplast via the ALMT1 could chelate Fe^3+^ and thus promote Fe accumulation. STOP1 and WRKY33 increase ALMT1 transcription under low-P conditions. SIZ1 downregulated the signaling of STOP1. The accumulation of STOP1 protein is inhibited by ALS3-STAR1 modules. It is possible that apo-Fe may be stored in the vacuole via VIT, but further confirmation is required to determine whether this pathway is present in all plants.

LOW PHOSPHATE ROOT 1 (LPR1) protein is considered to be a Fe-dependent P sensor that is a multicopper oxidase (Figure 2). It catalyzes the oxidation of Fe^2+^ to Fe^3+^ in the extracellular space, which is critical for Fe transport and homeostasis in plants [101,102]. LPR1 controls root development in P-deficient plants by modulating the accumulation of Fe in apoplast (apo-Fe) in the meristematic and elongation zones of the PR [96,98,103]. The aggregation of apo-Fe resulted in the formation of reactive oxygen species (ROS) and the deposition of callose, which promoted cell wall stiffening in the elongation zone [97,98]. This, in turn, led to the closure of plasmodesmata (PD), obstruction of intercellular transport pathways and inhibition of root cell proliferation, ultimately suppressed PR elongation [96,97,104]. Several processes have been reported to regulate or co-work with LPR1 under low-P conditions (Figure 2). *PHOSPHATE DEFICIENCY RESPONSE 2* (*PDR2*), encoded a single P5-type ATPase protein and expressed in endoplastic reticulum, restrained the activity of LPR1, and LPR1-PDR2 module was vital for deposition of Fe and callose in apoplast under low-P conditions [92,96]. The bZIP family transcription factor ELONGATED HYPOCOTYL5 (HY5) activated *LPR1* expression in *Arabidopsis* roots under P deficiency, resulting in the accumulation of apo-Fe [105]. In addition, HISTONE DEACETYLASE COMPLEX1 (HDC1) might interact with HISTONE DEACETYLASE 6 (HDA6) and be recruited to the LPR1 locus via a chromatin remodeler BRAHMA (BRM), thus suppressed *LPR1* transcription. Consequently, BRM, HDC1, and HDA6 negatively regulate the suppression of PR growth under low-P conditions [106,107].

Malate is a vital component in the regulation of apo-Fe in roots under Pi starvation conditions. Under low Pi conditions, the presence of Fe induced the accumulation of SENSITIVE TO PROTON RHIZOTOXICITY 1 (STOP1) in the nucleus, which thereby activated the expression of *ALUMINUM-ACTIVATED MALATE TRANSPORTER 1* (*ALMT1*) and elevated internal malate concentration [97,98,108]. Following oxidizing by LPR1, Fe^3+^ could form complexes with malate and suppress the reduction to Fe^2+^, thereby limiting Fe uptake by cells, further facilitating Fe deposition in the apoplast (Figure 2) [91,97,98]. In *Arabidopsis*, several pathways have been identified to regulate the STOP1-ALMT1 module under P-deficient conditions (Figure 2). As upstream proteins of STOP1, ALUMINUM SENSITIVE 3 (ALS3) interacted with SENSITIVE TO ALUMINUM RHIZOTOXICITY 1 (STAR1) to form an ABC transporter complex on the tonoplast membrane, which inhibited the accumulation of STOP1 protein in the nucleus, thus decreased the malate level [100,108,109]. SIZ1, a SUMO E3 ligase, negatively modulated STOP1 signaling to regulate Fe accumulation and hydroxyl radical production under P deficiency [110,111]. Additionally, the transcription factor WRKY33 promoted the accumulation of Fe^3+^ in root tips by modulating *ALMT1* expression, thus inhibited PR growth under low-P conditions [112].

Recently, the homeostasis of Fe, particularly Fe reduction in the apoplast, has attracted increasing attention (Figure 2). CYTOSOLIC ROOT REDUCTION (CRR) is a membrane protein that catalyzes the conversion of Fe^3+^ to Fe^2+^. Under low-P conditions, the *crr* mutant increased apo-Fe in the root meristem and shortened PR length compared to the wild type. Conversely, overexpression of CRR reduced apo-Fe deposition and alleviated the suppression of PR growth [8]. HYPERSENSITIVE TO LOW P1 (HYP1), a metalloreductase, is found to be upregulated in the proximal zone of the root apical meristem under P deficiency, which regulated Fe-dependent PR growth [113]. In addition, *PHOSPHATE-DEFICIENCY SENSITIVE1* (*PDE1*) encoded a hydroxyphenylpyruvate reductase, which was downregulated in response to P deficiency, concurrently enhancing Fe accumulation in the PR [114]. Collectively, the modulation of the oxidizing and reducing processes of apo-Fe represents a crucial pathway for regulating PR growth under P starvation.

It is notable that the Fe translocation could mitigate the accumulation of apo-Fe under low-P conditions. In contrast to *Arabidopsis*, the PR of rice is lengthened in P deficiency. This may be related to the upregulation of VIT, which leads to the sequestration of Fe into vacuoles (Figure 2) [88]. Nevertheless, further investigation is required to ascertain whether other plants exhibit the similar capacity for self-regulation, enabling themself to reduce Fe accumulation under P-deficiency. Furthermore, in response to prolonged P deficiency, root meristems were engaged in cell differentiation to prevent the accumulation of excess Fe and callose in apoplast. This process is mediated by CLAVATA3/ENDOSPERM SURROUNDING REGION 14 (CLE14). In P deficiency, Fe accumulation in the root meristem led to the expression of CLE14 in the proximal meristematic region. The receptors CLAVATA2 (CLV2) and CLV2/PEP1 RECEPTOR 2 (PEPR2) were capable of sensing CLE14, which in turn triggered differentiation in the root meristem to prevent long-term damage of PR induced by low-P [115].

Root hair (RH) serve to enhance the surface area of root, thereby facilitate the acquisition of nutrients. The promotion of RH development represents a strategy employed by plants to adapt to low-P environments, which also necessitates the maintenance of Fe homeostasis [6]. In *Arabidopsis* under P deficiency, the formation of RH was contingent upon an optimal Fe concentration, both excessive and deficient Fe supplementation impeded RH development [116]. PHOSPHATE RESPONSE (PHR), the core transcription factor of the P starvation response, regulated Fe-mediated RH development in low-P conditions. This was primarily accomplished by directly regulating expression of Fe-homeostasis related gene *FERRITIN1* (*FER1*), which facilitated the removal of excess Fe [117,118]. Furthermore, alterations in Fe concentrations under low-P condition influenced vesicle trafficking, which in turn affects PIN-FORMED 2 (PIN2) recycling, further promoted auxin transport to affect PHR1 expression, ultimately facilitated RH growth [6]. HEMERYTHRIN MOTIF-CONTAINING REALLY INTERESTING NEW GENE AND ZINC FINGER PROTEINS (HRZs), a potential Fe sensor, was downregulated in low-Fe condition and triggered the degradation of the PHR2 protein [119], suggested that HRZs interacts with PHR2 to coordinate P and Fe homeostasis in rice.

### 3.3. The Exudates Metabolism Response to Fe-P Interaction

In response to nutrient stress, plants secrete secondary metabolites internally to modulate the process of nutrient deficiency, and externally to activate insoluble nutrients in the rhizosphere [120]. Similar to microorganisms, organic acids are vital secondary metabolites secreted by plants under P or Fe deficiency [121,122]. In Fe-deficient conditions, the exudation of malate and citrate is elevated by low-P, and the exogenous application of malate and citrate effectively alleviates Fe and P deficiencies [85]. After secreting into the rhizosphere, organic acids mobilize Fe and P under acidic conditions via chelation with Fe and competing for adsorption sites of Pi [123,124,125,126]. Among organic acids from root exudates, citrate exhibits the greatest capacity for activating soil P, followed by oxalate, malate, acetic acid, and succinic acid [127,128]. In both Fe-limited and P-limited condition, malate and citrate are secreted in abundance into the rhizosphere [129,130], suggesting that these two organic acids may be crucial root exudates for activating insoluble Fe-P in the soil.

The redox substances secreted by plants of strategy I are crucial in regulating the availability of P in the soil. For example, coumarins from root exudates are not only induced by Fe deficiency but also upregulated by P deficiency. In the rhizosphere, coumarins facilitate the reduction of Fe^3+^ to release P from Fe-P complexes [131,132], which also contributes to Fe accumulation induced by Pi starvation [80]. The secretion of coumarins is orchestrated by the MYB63 transcription factor through the PHOSPHATE DEFICIENCY RESPONSE9 (PDR9) pathway under Fe and P combined deficient conditions [133]. However, recent studies have demonstrated that low-P is conversely found to downregulate the secretion of several kinds of coumarins under Fe-limited conditions [134,135]. These discrepancies in coumarin secretion induced by low-P may be related to the distinct types of coumarins having varying degrees of efficiency in activating soil Fe and P, as well as in their metabolism within plants. This is evidenced by the upregulation of esculin, esculetin and scopoletin, but downregulation of dihydroxyscopoletin under P deficiency [132]. In addition to coumarins, flavonoids secreted by lupin roots have been shown to mobilize insoluble Fe-P [136]. Although multiple redox-active root exudates have been validated for regulating plant Fe nutrition [75,76,137,138,139], further investigation is required to determine whether they can activate P by releasing Fe.

The phytosiderophore secreted by strategy II plants under low-Fe conditions is regulated by external P levels. In Fe-limited environments, low-P enhances early growth and Fe accumulation in the shoot of barley, but suppresses the release of mugineic acids [140]. The inhibition in phytosiderophore secretion may be associated with alleviating Fe-deficient chlorosis. Nevertheless, the role of phytosiderophore in activating insoluble Fe-P complexes in the rhizosphere, and the direct influence of P levels on the biosynthesis as well as secretion pathways of phytosiderophore remains inadequately supported by evidence.

The community and function of microorganisms in the rhizosphere can be reshaped by root exudates, which act as nutrients and energy sources [141]. Recruited beneficial bacteria have been demonstrated to modulate the P-Fe interaction in plants. A strain of *Pseudomonas putida* enhanced expression of two Pi starvation-induced genes and inhibited PR growth by accumulating Fe in the roots of *Arabidopsis* [142]. Furthermore, *P. putida* was observed to alleviate chlorosis in *fit1* and *irt1-1* mutants, and suppress the expression levels of *IRT1* in the wild-type plants [142]. These evidences suggests that *P. putida* may regulate the utilization of P via modulating P signaling in plants, but influence Fe uptake through special mechanism, such as siderophore secretion. Although plants occupy advantageous positions in activating insoluble Fe-P in soil compared to rhizobacteria [143], elucidating the modulatory mechanisms of microbial composition by root exudates and plant growth by beneficial bacteria could provide new insights into the efficient utilization of nutrients mediated by microbe-plant interactions in the rhizosphere. It is also crucial to acknowledge that endogenous secretions also exert regulatory effects on endophytic microorganisms, which may subsequently influence the responses to Fe and P deficiencies, including apo-Fe accumulation, P and Fe signal transduction. This represents a significant avenue for future research.

### 3.4. The Fe-Influenced Hormone Metabolism Response to Low-P

The metabolism of hormones is vital for plants in response to nutrient deficiencies [144,145]. In adaption to low-P and low-Fe environments, the metabolisms of primary hormones are significantly regulated in plant, including auxins, gibberellins, ethylene, and brassinosteroids. For instance, Fe deficiency suppresses gibberellin in roots, resulting in the accumulation of DELLA growth-inhibitory factors in the meristematic and elongation zones, thereby inhibiting the elongation of the PR [146]. Although a similar process has also been observed in P-deficient plants [147], further investigation is required to demonstrate whether gibberellin metabolism is related to P-Fe interaction in plants. Fe and P deficiencies also regulate the metabolisms of ethylene and auxins. In low-Fe conditions, ethylene and auxins synergistically inhibit root elongation [148,149,150]. In *Arabidopsis* roots, enhanced ethylene signaling promotes auxin synthesis in the root tips, which is associated with the PR development under low-P conditions [151], suggesting that ethylene and auxin interaction might participate in responses mediated by apo-Fe in P deficiency.

The functions of brassinosteroids (BR) have attracted considerable attention in recent years, mainly due to its capacity to alleviate the adverse effects of biotic and abiotic stresses on plants [152,153]. Furthermore, BR is involved in regulating the responses of plants to low-Fe and low-P conditions. BRASSINAZOLE RESISTANT 1 (BZR1), a key transcription factor in the BR signaling pathway, controls Fe accumulation in the root elongation zone by suppressing *LPR1* expression, thus prevents PR inhibition in P deficiency (Figure 2). Conversely, under low-Fe conditions, activation of BR signaling may lead to cell wall relaxation, promoting cell elongation and root growth [99]. A suppressor of BR signaling, *BRI1 KINASE INHIBITOR 1* (*BKI1*), is downregulated by low-Fe and upregulated by low-P to modulate root development [99]. In addition, BR interacts with ethylene to regulate plant responses to Fe deficiency. 24-epibrassinolide (EBR), a member of the BR family, has been observed to increase the activity of the FRO via the inhibition of ethylene production in high-Fe seedlings, while reduce its activity in low-Fe seedlings [154], whether this process is related to external P levels requires further investigation.

### 3.5. The Photosynthesis Affected by Fe-P Interaction

Fe and P are essential components of photosynthetic enzymes, and are vital for chlorophyll synthesis. Deficiencies in either Fe or P severely impede photosynthesis [155]. The inhibition of photosynthesis induced by Fe-deficiency is dependent on the P status. Under high-P conditions, plants are more prone to exhibit Fe-deficiency chlorosis and genes related to chloroplast synthesis and photosynthesis are downregulated, whereas the chlorosis is mitigated by reducing the P level [156]. In P starvation, Fe is one of the most accumulated metals in chloroplasts, and genes related to Fe homeostasis, including *NICOTIANAMINE SYNTHASE 3* (*NAS3*) and *FER1*, are upregulated in *Arabidopsis* [79,157], implying that Fe distribution is essential for the photosynthetic response to low-P conditions. In *Arabidopsis*, *PHOSPHATE CO-TRANSPORTER 4;4* (*PHT4;4*), encodes an ascorbate transporter in chloroplasts, interacted with a transcription factor bZIP58, resulted in an alleviation in the decline of photosynthesis induced by low-Fe and low-P deficiency [158]. Under Fe deficiency, P deprivation led to an upregulation of *bZIP58* expression, thereby enhanced the transcription levels of nucleus-encoded photosynthetic genes. Concurrently, bZIP58 promoted the synthesis of ascorbate (AsA) via induction of *VITAMINC4* (*VTC4*) expression, then increased ascorbic acid in the chloroplast mediated by PHT4;4. AsA prevented the accumulation of ROS, which in turn maintained the expression of *bZIP58* and its downstream photosynthetic genes [158].

## 4. Perspectives

In the past few decades, the Fe-P interactions in plants and soil have been extensively studied, yet the comprehensive effects of this interaction in conjunction with plants and soil remain to be elucidated. In order to explore the Fe-P interaction in soil, root, and leaf, a multi-dimensional interactome could be constructed, including genomics, transcriptomics, proteomics, metabolomics and microbiomics. In addition, the endophyte has been demonstrated to enhance the utilization efficiency of nutrients in plants. It would be of interest to determine whether the endophyte could influence the homeostasis and signaling of Fe and P in plants, particularly with regard to regulating apo-Fe status in roots under low-P conditions. Furthermore, given the intricate relationship between P and Fe in plants and soil, it is important to develop and apply P and Fe fertilizers in a manner that is appropriate for the specific conditions. The application of beneficial microorganisms and functional substances, derived from the soil-plant interaction process, to the dissolution of insoluble Fe-P complexes could be a significant pathway to the synergistic achievement of efficient Fe and P in acidic soil.

## Data Availability

There is no new data in this work.
